# [^68^Ga]-DOTATOC-PET/CT for meningioma IMRT treatment planning

**DOI:** 10.1186/1748-717X-4-56

**Published:** 2009-11-18

**Authors:** Barbara Gehler, Frank Paulsen, Mehmet Ö Öksüz, Till-Karsten Hauser, Susanne M Eschmann, Roland Bares, Christina Pfannenberg, Michael Bamberg, Peter Bartenstein, Claus Belka, Ute Ganswindt

**Affiliations:** 1 Department of Radiation Oncology, University of Tübingen, Hoppe-Seyler-Str 3, 72076 Tübingen, Germany; 2 Department of Radiation Oncology, LMU München, Marchioninistr. 15, 81377 München, Germany; 3 Department of Nuclear Medicine, University of Tübingen, Hoppe-Seyler-Str 3, 72076 Tübingen, Germany; 4 Department of Neuroradiology, University of Tübingen, Hoppe-Seyler-Str 3, 72076 Tübingen, Germany; 5 Department of Radiology, University of Tübingen, Hoppe-Seyler-Str 3, 72076 Tübingen, Germany; 6 Medizinisches Versorgungszentrum Nuklearmedizin, Marienhospital Stuttgart, Böheimstr 37, 70199 Stuttgart, Germany; 7 Department of Nuclear Medicine, LMU München, Marchioninistr 15, 81377 München, Germany; 8 Department of Radiology, University Hospital Basel, Petersgraben 4, CH 4031 Basel, Switzerland

## Abstract

**Purpose:**

The observation that human meningioma cells strongly express somatostatin receptor (SSTR 2) was the rationale to analyze retrospectively in how far DOTATOC PET/CT is helpful to improve target volume delineation for intensity modulated radiotherapy (IMRT).

**Patients and Methods:**

In 26 consecutive patients with preferentially skull base meningioma, diagnostic magnetic resonance imaging (MRI) and planning-computed tomography (CT) was complemented with data from [^68^Ga]-DOTA-D Phe^1^-Tyr^3^-Octreotide (DOTATOC)-PET/CT. Image fusion of PET/CT, diagnostic computed tomography, MRI and radiotherapy planning CT as well as target volume delineation was performed with OTP-Masterplan^®^. Initial gross tumor volume (GTV) definition was based on MRI data only and was secondarily complemented with DOTATOC-PET information. Irradiation was performed as EUD based IMRT, using the Hyperion Software package.

**Results:**

The integration of the DOTATOC data led to additional information concerning tumor extension in 17 of 26 patients (65%). There were major changes of the clinical target volume (CTV) which modify the PTV in 14 patients, minor changes were realized in 3 patients. Overall the GTV-MRI/CT was larger than the GTV-PET in 10 patients (38%), smaller in 13 patients (50%) and almost the same in 3 patients (12%). Most of the adaptations were performed in close vicinity to bony skull base structures or after complex surgery. Median GTV based on MRI was 18.1 cc, based on PET 25.3 cc and subsequently the CTV was 37.4 cc. Radiation planning and treatment of the DOTATOC-adapted volumes was feasible.

**Conclusion:**

DOTATOC-PET/CT information may strongly complement patho-anatomical data from MRI and CT in cases with complex meningioma and is thus helpful for improved target volume delineation especially for skull base manifestations and recurrent disease after surgery.

## Introduction

Meningiomas represent about 20% of all intracranial brain tumors and are therefore the most frequent nonglial brain tumors in adults with a clear predomination in women (f/m 2:1) [1]. More than 90% are histological benign of mesodermal origin arising from the arachnoid meninges of the brain and are slow-growing with a low proliferation index. Atypical or malignant histology is rare and often requires multimodal treatment caused by increased local relapse.

Particularly meningiomas of the skull base are difficult to treat due to their close relation to critical structures like brainstem, major vessels and cranial nerves.

Although surgical resection of meningioma is the preferred treatment approach, ionizing radiation is a highly effective treatment modality. After complete surgical resection long-term recurrence-free survival can be achieved up to 93 and 80% after 5 and 10 years, respectively. Without total removal the recurrence-free survival is inferior, up to 65 and 45% or worse after 5 and 10 years, respectively [1-4]. Adjuvant radiotherapy (RT) can improve local tumor control and overall survival after incomplete surgical resection [5-8]. Similar to resection, radiotherapy alone offers 5-year local control above 90% [9,10]. However, the surrounding tissue and the benign histology mandate extreme precision during treatment planning in order to minimize the risk of side effects. Therefore stereotactic fractionated treatment protocols comprise the standard radiotherapy approach. Similar to numerous different malignancies treated with intensity-modulated RT [11-13], recently several IMRT based protocols for meningiomas have been issued offering even higher target volume conformity and improved normal tissue protection [14-19]. With increasing conformity the need for accurate target volume delineation is of outmost importance. Of special importance is the fact that target volume definition after single or repeated surgical intervention is frequently hampered by artifacts. In general the use of highly conformal treatment techniques mandates improved pretherapeutic imaging. In this regard, positron emission tomography (PET) based techniques as well as other functional imaging modalities including SPECT-CT or also MRI enter the routine in radiation oncology [20-26].

Up to now treatment planning was mainly based on combinations of contrast enhanced CT and MRI. Especially after repetitive surgery and in case of an infiltrative growth pattern these imaging modalities have their limitations.

Meningioma cells strongly express somatostatin receptor subtype 2 (SSTR 2) which offers an additional positron emission tomography (PET) based imaging for tumor delineation with the somatostatin-receptor ligand [^68^Ga]-DOTA-D Phe^1^-Tyr^3^-Octreotide (DOTATOC) [27]. DOTATOC-PET/CT shows a high meningioma to background ratio which can be used to improve target volume definition prior to IMRT [28,29].

To document the value of [^68^Ga]-DOTATOC-PET/CT for treatment planning of complex meningiomas preferentially of the skull base we retrospectively analyzed a series of patients in whom CT/MRI based treatment planning was complemented by [^68^Ga]-DOTATOC-PET/CT.

## Patients and Methods

### Patients

26 consecutive patients with preferentially skull base meningiomas received diagnostic MRI, RT planning CT and additional [^68^Ga]-DOTATOC-PET/CT prior to fractionated stereotactic IMRT between 2007 and 2008 in our institution. 20 meningiomas were located at the skull base, one was an optic nerve sheath meningioma. Median age at treatment was 59.5 years (range 28-82 years). The male/female ratio was 3/23, median Karnofsky performance score was 90% (range 70-100%). 19 of the 26 patients underwent surgical treatment or extended biopsy, 13 once, five twice and one woman for three times before start of RT. The pathological examination revealed a World Health Organization (WHO) grade I meningioma in 14 and a WHO grade II tumor in four patients. One young patient with a WHO grade II meningioma received a prior prophylactic radiation of the brain with a cumulative dose of 18 Gy due to the therapy regime of a hematological disorder 26 years before. Seven patients received IMRT as primary treatment without proven histology because biopsy was concluded to be infeasible. In these cases diagnosis of meningioma was based on CT and MRI offering typical radiologic characteristics of a benign meningioma. Characteristics of the patients are listed in Table 1.

**Table 1:** Patients characteristics

Patients treated with IMRT	n = 26
Patients (female/male)	23;3
Karnofsky Performance Scale (median/range) [%]	95 (70-100)
Age (median/range) [years]	60 (28-82)
Tumor site	
Olfactorius nerve	5
Optic nerve sheath	1
Sphenoidal	4
Cavernous sinus	2
Petroclival/clival	5
Frontoparietal/-basal	2
Sphenoorbital	2
Parasagittal/falx	3
Infratentoriell	1
Convexity	1
Surgery/biopsy	19
resection for 1/2/3 times	13;5;1
Histology/WHO grading	
WHO I	14
WHO II (atypical meningioma)	4
Tissue lost	1
Unknown (diagnosis based on MRI, CT)	7
Postoperative period until initiation of radiation (median/range) [months]	56.1 (3-249)

### [^68^Ga]-DOTATOC-PET/CT

Imaging was performed using a dedicated PET/CT scanner (Biograph 16 HiRez; Siemens Medical Solutions, Erlangen, Germany).

Forty minutes after intravenous injection of 150 MBq [^68^Ga]-DOTATOC the combined examination commenced with a topogram to define the PET/CT examination range (2 fields of view (FOV)). Non-contrast CT scans were performed firstly for attenuation correction of PET data and for anatomic correlation. Subsequently the PET scan was done acquiring static emission data for 4 minutes per FOV.

PET images were reconstructed by using an iterative algorithm (ordered-subset expectation maximization: 4 iterations, 8 subsets). Non-enhanced CT data were reconstructed with a slice thickness of 5 mm (axial) and an increment of 5 mm.

The reconstructed PET, CT and fused images were displayed on the manufacturer's workstation (e-soft, Siemens Medical Solutions) in axial, coronal and sagittal planes with a resolution of 128 × 128 pixels for the PET and 512 × 512 pixels for the CT images.

The fused PET/CT images were evaluated by two experienced nuclear medicine experts and two experienced radiologists in consensus. For all detected meningiomas the standardized uptake value (SUV) was calculated using the region of interest (ROI, 50% isocontour) method and was corrected for weight.

### Treatment planning and target volume definition

IMRT treatment planning was primarily based on diagnostic MRI data and was secondarily complemented by the information from [^68^Ga]-DOTATOC-PET. Additionally all patients routinely had a neuroophthalmological and endocrinological examination and an audiometry. RT planning was performed on a 3D-data set generated from 3 mm CT scans in treatment position. For immobilization of the head an individual thermoplastic head mask fixation was used. Image fusion of diagnostic MRI, RT planning CT and PET/CT as well as target volume delineation was done with OTP-Masterplan^® ^package (Theranostic GmbH, Solingen, D). The CT planning images in mask fixation were fused with the CT images derived from PET/CT (CT to CT, additionally CT to diagnostic MRI) using the automatic matching algorithm stored in the OTP-Masterplan^® ^system. As being initially linked to the combined PET/CT images the raw PET data did not require a separate image fusion.

For gross tumor volume (GTV) delineation the initial macroscopic tumor volume definition was based on MRI findings and RT planning CT information only (GTV-MRI/CT). Subsequently the PET positive tumor lesions were defined by the same therapist (GTV-PET). The GTV-MRI/CT as well as the GTV-PET was counterchecked by an advanced neuroradiologist or rather nuclear medicine physician. MRI data were complemented by DOTATOC-PET findings and additional clinical information (particularly including potential areas of microscopic tumor growth) with a resulting CTV. Finally the CTV was expanded with an overall safety margin of 4 mm to the PTV.

For IMRT treatment planning organs at risk (brainstem, optical nerves, chiasm, lens, internal ear and hippocampus) were outlined. Irradiation was performed as EUD (equivalent uniform dose)-based IMRT, using the Hyperion^® ^software package. Three-dimensional dose distributions were calculated and optimized via Monte Carlo dose calculation using a multileaf collimator (leaf width: 4 mm at isocenter). The purpose of treatment planning was to cover the 95% isoline by the PTV. The dose prescription was 54 Gy in total with a daily fraction dose of 1.8 Gy, 5 times a week. Patient positioning was verified by cone beam CT imaging every day in the first week of irradiation and afterwards twice a week.

### Quantitative analysis of tumor volumes

For quantification of target volume changes based on the PET findings we evaluated both the tumor volumes (GTV-MRI/CT and GTV-PET) and intersection areas between the GTV-MRI/CT and GTV-PET (Intersection-GTV-MRI/CT/PET). For both modalities we computed the increase in [cc] with respect to the intersection area (Increase-MRI/CT vs. Intersection, respectively Increase-PET vs. Intersection). These areas are those, which are visible in one target volume only. Finally the ratios between the increased volumes with respect to the GTV-MRI/CT were assessed. In order to report these volume values for the whole patient collective pure descriptive statistics (mean, standard deviation, median, maximum, minimum) were used.

## Results

### IMRT

A median treatment dose of 53 Gy (range 51.2-57 Gy) could be achieved. IMRT was submitted with a 6/15 MV linear accelerator (Elekta Synergy SBM XVI) and was carried out on average with 8 beams (range 6-10) and 41 segments (range 17-70).

### Target volume definition by MRI/CT

The GTV-MRI/CT included the macroscopic tumor visible in the planning CT and contrast-enhanced T1-weighted MRI. All meningiomas could be delineated on MRI and CT. For the GTV-MRI/CT no safety margin was defined. Median GTV-MRI/CT was 18.1 cc (mean: 27.5; range 1.2-79.5 cc).

### Target volume definition by [^68^Ga]-DOTATOC-PET

All 26 patients displayed a pronounced SSTR 2 tracer retention within the meningioma. In addition to a strong signal in three patients there were distant small spots without a morphologic correlate in the cranial MRI, which were not included in the GTV-PET.

There were several DOTATOC-PET positive lesions beyond the cranium without any further suspicious tumor detection in an additional CT or MRI examination.

Target volume definition based on [^68^Ga]-DOTATOC-PET (GTV-PET) included the tumor volume with an intense tracer uptake, all 26 meningiomas showed a high tumor-to-background contrast. For the target volume definition the windowing of the DOTATOC-PET was determined by the optimal matching between the PET-positive areas and the viewable tumor margins determined by CT/MRI. The physiological signal of the bony skull and the air-filled nasal cavity was masked out via windowing; neither a SUV cut-off nor a safety margin was defined. In general, the differentiation between the pituitary gland and adjacent located tumor manifestations is mostly sophisticated caused by the SSTR 2 expression of the gland itself. In cases with PET positive tumor manifestations nearby the pituitary gland, they were included completely if it was not possible to distinguish gland from tumor manifestation. The median GTV-PET was 25.3 cc (mean: 33.5 cc; range 0.6-106.1 cc).

### Correlation of GTV-PET and GTV-MRI/CT - Multimodal target volume definition

**Figure 1:**
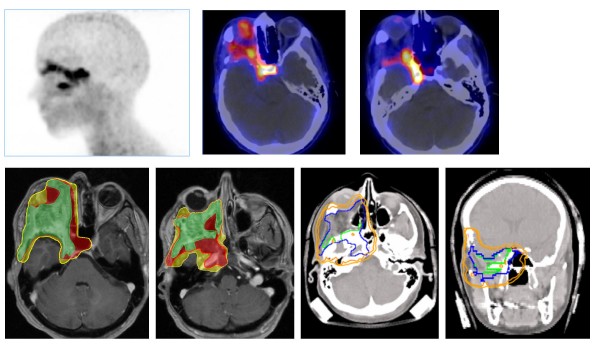
**Large skull base meningioma with orbital invasion and close relation to the sella turcica region, [68Ga]-DOTATOC-PET (top left)/CT image fusion (top right)**. Physiological tracer uptake of the pituitary gland. CTV/GTV contours (below left): red = GTV-PET; green = GTV-MRI/CT; yellow = CTV, CTV enlargement by GTV-PET. Dose distribution with enclosing 90% PTV isoline.

**Figure 2:**
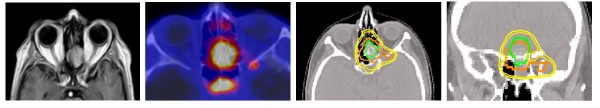
**Recurrence of olfactory's meningioma (MRI left)**. [68Ga]-DOTATOC-PET/CT image fusion with small distant lesion at the left dorsal orbital bone and physiological tracer uptake of the pituitary gland. Dose distribution (right) with inclusion of the small distant lesion and enclosing 90% PTV isoline.

In 17 of 26 patients DOTATOC-PET data led to additional information concerning the tumor extension (examples Figures 1, 2). Overall the GTV-MRI/CT was larger than the GTV-PET in 10 patients, smaller in 13 patients and almost the same in three patients (< = 0.7 cc deviation). Among the 13 patients with a larger GTV-PET than GTV-MRI/CT there were three patients with an inclusion of the pituitary gland region caused by difficult discrimination gland from tumor manifestation.

In 14 cases there were major changes of the clinical target volume (CTV) based on PET findings (10 enlargements, two reductions and in two cases areas of target volume enlargement as well as reduction). Minor changes (only changes at the borders of the target volume without affection of a new anatomical area) were seen in three patients (three enlargements). Three cases showed a pronounced enlargement of the CTV in the postoperative situation based on enclosure of the PET positive resection hole. Exemplarily, in one patient the conventional MRI imaging showed no residual tumor growth after resection of an olfactory's meningioma, whereas the additional PET data revealed active tumor mass in the nasal cavity and in the ethmoidal sinus. Therefore a clear enlargement of the CTV (GTV-MRI/CT 13.6 cc; GTV-PET 18.2 cc; CTV 19.6 cc) resulted. In one case there was a remarkable enlargement of the CTV caused by a PET positive osseous lesion which could not clearly be seen on MRI and CT imaging (GTV-MRI/CT 69.3 cc; GTV-PET 94 cc; CTV 99 cc). In 9 of 26 patients DOTATOC-PET delivered no supplementary information regarding tumor extension known from MRI and CT. The median CTV as a summation of the GTV-MRI/CT and the GTV-PET without a safety margin was 37.4 cc (mean 42.2 cc; range 1.3-143.2 cc). With the exception of three patients in the group of patients with additional information from the DOTATOC-PET the CTV was always larger than each single of the correlating GTV (PET and MRI/CT). The PTV was created from the CTV with an overall safety margin of 4 mm. Median PTV was 78.3 cc (mean: 92.3 cc; range 6.8-227.7 cc).

The median intersection volume of the GTV-MRI/CT and the GTV-PET was 13.4 cc (mean 21.3 cc). The median volume increase based on the PET findings compared to the intersection was 6.1 cc (mean 12.2 cc ± 13.3 cc), based on the MRI and planning-CT data 5.7 cc (mean 6.2 cc ± 4.6 cc). Hence the increase is approximately the same for both defined GTVs. The median ratio of the overall MRI-positive but PET-negative volume to the GTV-MRI/CT was 0.28 (mean 0.33 ± 0.21; range 0.02-0.75). The percentage of enlargement over the GTV-MRI/CT based on DOTATOC-PET was 0.31 (mean 1.03 ± 2.8; range 0-14.36, after removing one patient with a PET-tracer uptake in the resection hole without a visible tumor growth in the MRI, the median ratio was 0.3 (mean 0.49 ± 0.68; range 0-3.1) (Table 2). We conclude that about 30% of the GTV-MRI/CT display no PET-tracer uptake and vice versa the volume outside the GTV-MRI/CT with PET-tracer uptake not being visible in the MRI/CT images has a volume approximately of 30% of the GTV-MRI/CT.

**Table 2:** Treatment characteristics, target volumes

	Median	Maximum	Minimum	SD	Mean
GTV-MRI/CT [cc]	18,1	79,5	1,2	23,5	27,5
GTV-PET [cc]	25,3	106,1	0,6	29,1	33,5
CTV [cc]	37,4	143,2	1,3	34,7	42,2
Intersection-GTV-MRI/CT/PET [cc]	13,4	78,2	0,3	21,5	21,3
Increase-MRI/CT vs Intersection [cc]	5,7	15,5	0,8	4,6	6,2
Increase-PET vs Intersection [cc]	6,1	48,8	0	13,2	12,2
Ratio Increase-MRI/CT to GTV-MRI/CT	0,28	0,75	0,02	0,21	0,33
Ratio Increase-PET to GTV-MRI/CT	0,31	14,36	0,00	2,80	1,03

## Discussion

A wide range of publications has documented the value of external beam radiation for the treatment of meningioma. However clinical practice is more likely to show that only those cases suffering from complex meningioma are referred to radiotherapy. This included patients with relapse after surgery, large tumors or complexly growing tumor. Thus the treating physician is frequently faced with the dilemma to spare as much of critical normal tissue without missing gross tumor. The use of highly conformal treatments including IMRT even increases the need for optimal target volume delineation.

In the present study we evaluated the value of the [^68^Ga]-DOTATOC-PET for treatment planning of intracranial complexly shaped meningiomas. Up to now the follow up time in our cohort is all too short to give some information about local control after IMRT treatment. However, our data show clearly that the use of [^68^Ga]-DOTATOC-PET improved target volume delineation in a larger proportion of our patients schedule for an IMRT based radiation approach when compared to MRI based planning alone. Particularly bony lesions or direct bone infiltration by adjacent meningioma tissue were more likely to be detected with PET. Basically we found a geographical miss in 50% of the patients and - on the other hand - were able to reduce the CTV in 38% of the patients. When compared to other observations using [^68^Ga]-DOTATOC-PET, ^11^C-Methionine [30,31] or ^18^F-Tyrosine [32], similar ranges were reported in the term of PET scanning offering additional information. In this regard Milker-Zabel reported relevant information in 19 out of 26 patients using DOTATOC [33], Astner reported additional information in 29 out of 32 patients [30] and Rutten reported changes in 6 out of 13 lesions in 11 patients using ^18^F-Tyrosine [32]. In our series in 17 out of 26 patients PET scanning offered relevant complementary information.

When one analyzes the pattern of changes in more detail, Milker-Zabel and Rutten reported larger proportions of potential geographical misses avoided by PET scanning (38% in both studies) [32,33]. This is in accordance with our findings where the CTV was increased after inclusion of the PET data (50%). In contrast, the study by Astner reported a larger proportion of GTV/PTV reductions after inclusion of ^11^C-Methionine-PET data when compared to MRI scanning alone (75%) [30].

The reasons for these differences are not readily deducible from the reported data. However, it may be speculated that the inherent bias of patient selection and strategies employed for MRI-GTV definition may be the underlying reason. This assumption is supported by the fact that at least comparable volumes were treated in all three studies excluding the possibility that differences in tumor volume are responsible for the differences in target volume changes.

An important consideration in this context is the open question if there is s SUV-threshold to define the GTV-PET. In our study for the target volume definition the windowing of the DOTATOC-PET was determined by the matching between the PET-positive areas and the viewable tumor margins determined by CT/MRI. The physiological signal of the bony skull and the air-filled nasal cavity was masked out via windowing. Although a SUV-threshold would be helpful for the GTV-DOTATOC-PET delineation in meningiomas, up to know clear evidence for a SUV cut-off is missing. Astner et al. [34] reported an interesting phantom study in 11 patients with glomus tumors and revealed that a value of 32% of the maximum standardized uptake was an appropriate threshold for tumor delineation. At the moment we do not have this information for meningiomas in DOTATOC-PET imaging. However, in regard to IMRT planning for meningiomas special biological characteristics of microscopic tumor growth have to be taken into account especially for CTV delineation. Hence in our opinion we have to be cautious in reducing target volumes along an experimental SUV-threshold alone.

From the data currently available it seems that either [^68^Ga]-DOTATOC, ^11^C-Methionine or ^18^F-Tyrosine are useful tracers for target volume definition in patients with meningioma. Up to now there is no clear evidence available supporting the superiority of any of the given tracers.

As stated above, meningiomas particularly show high levels of somatostatin receptor expression (SSTR2) resulting in a high tracer uptake. The usefulness of [^68^Ga]-DOTATOC-PET for a distinction of meningioma from other brain tumors has been well documented [35-37]. In several disorders including metastasis or glioma Methionine or Tyrosine may produce false positive results. However, amino acid tracers like Methionine or Tyrosine are markers of amino acid transport and give some more information in regard to metabolic activity of several tumor tissues. At least Methionine-PET may help to judge the aggressiveness of meningioma since the uptake has been reported to correlate with the proliferative activity measured by the KI-67 index [38-40]. In our opinion for IMRT planning it seems reasonable to use the tracer with the highest inherent specificity which - by means of its mechanism of action - is [^68^Ga]-DOTATOC-PET.

A given disadvantage of DOTATOC is the fact that the pituitary gland is generally highly positive and limits the precision of target volume definition in this area.

Although PET/CT images have reached a considerable level of spatial discrimination the current technology does not allow for the visualization of microscopic tumor growth along the dural membranes. Thus it will be still necessary to add empirical margins to cover all areas.

## Conclusion

[^68^Ga]-DOTATOC-PET/CT information strongly complements image data from MRI and CT in cases with complex meningiomas of the skull base. In all meningioma patients a tracer uptake of the [^68^Ga]-DOTATOC was seen. Especially in patients with complex skull base meningioma or recurrent disease [^68^Ga]-DOTATOC offers important additional information. Therefore we would recommend the use of the [^68^Ga]-DOTATOC for GTV definition in all cases with complex meningioma.

Further evaluation with a larger number of patients seems to be justified and long-term follow-up is needed to evaluate the clinical impact.

## Abbreviations

cc: cubic centimetre; CT: computed tomography; CTV: clinical target volume; 3-D: three-dimensional; DOTATOC: [^68^Ga]-DOTA-D Phe^1^-Tyr^3^-Octreotide; EUD: equivalent uniform dose; f: female; FOV: field of view; GTV: gross tumor volume; IMRT: intensity modulated radiotherapy; m: male; MBq: megaBecquerel; MRI: magnetic resonance imaging; PET: positron emission tomography; PTV: planning target volume; ROI: region of interest; RT: radiotherapy; SPECT: single photon emission computed tomography; SSTR: somatostatin receptor; SUV: standardized uptake value.

## Competing interests

The authors declare that they have no competing interests.

## Authors' contributions

CB & UG planned, coordinated and conducted the study. MÖ, SE, RB & CP performed PET imaging. BG, T-KH & MÖ analyzed the PET and MRI imaging data. BG, UG, CB & FP analyzed the treatment planning data. BG, CB, PB & UG prepared the manuscript. Medical care was covered by BG, UG, CB, FP & MB. All authors read and approved the final manuscript.
